# A Review of Multiple Mitochondrial Dysfunction Syndromes, Syndromes Associated with Defective Fe-S Protein Maturation

**DOI:** 10.3390/biomedicines9080989

**Published:** 2021-08-10

**Authors:** Elise Lebigot, Manuel Schiff, Marie-Pierre Golinelli-Cohen

**Affiliations:** 1 Bicêtre Hospital, AP-HP, Biochemistry Department, 94275 Le Kremlin-Bicêtre, France; 2 Necker Hospital, AP-HP, Reference Center for Inborn Error of Metabolism and Filière G2M, Pediatrics Department, University of Paris, 75015 Paris, France; manuel.schiff@aphp.fr; 3 Inserm UMR_S1163, Institut Imagine, 75015 Paris, France; 4 Institut de Chimie des Substances Naturelles, CNRS, UPR 2301, Université Paris-Saclay, 91198 Gif-Sur-Yvette, France

**Keywords:** MMDS, Fe-S proteins, NFU1, IBA57, BOLA3, ISCA1, ISCA2

## Abstract

Mitochondrial proteins carrying iron-sulfur (Fe-S) clusters are involved in essential cellular pathways such as oxidative phosphorylation, lipoic acid synthesis, and iron metabolism. NFU1, BOLA3, IBA57, ISCA2, and ISCA1 are involved in the last steps of the maturation of mitochondrial [4Fe-4S]-containing proteins. Since 2011, mutations in their genes leading to five multiple mitochondrial dysfunction syndromes (MMDS types 1 to 5) were reported. The aim of this systematic review is to describe all reported MMDS-patients. Their clinical, biological, and radiological data and associated genotype will be compared to each other. Despite certain specific clinical elements such as pulmonary hypertension or dilated cardiomyopathy in MMDS type 1 or 2, respectively, nearly all of the patients with MMDS presented with severe and early onset leukoencephalopathy. Diagnosis could be suggested by high lactate, pyruvate, and glycine levels in body fluids. Genetic analysis including large gene panels (Next Generation Sequencing) or whole exome sequencing is needed to confirm diagnosis.

## 1. Introduction

**Figure 1:**
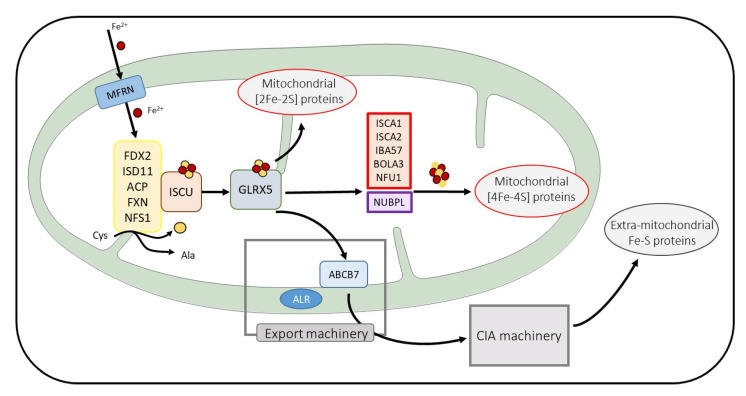
Schema of the main steps of Fe-S protein maturation. Only the mitochondrial ISC machinery components are detailed. Mitoferrin (MFRN) is involved in iron entry in mitochondria. Proteins in yellow frames are needed for cysteine desulfuration and [2Fe-2S] cluster de novo assembly on the ISCU. After its transfer on GLRX5, the [2Fe-2S] cluster is exported to the cytosol (for extra-mitochondrial Fe-S protein maturation) and is inserted to mitochondrial [2Fe-2S] proteins or is taken in charge by proteins essential for [4Fe-4S] cluster biosynthesis and the maturation of mitochondrial [4Fe-4S] containing proteins. Proteins involved in MMDS pathologies are framed in red.

Conserved across all kingdoms of life, iron-sulfur (Fe-S) clusters are ancient prosthetic groups. They are found in a variety of proteins involved in diverse essential cellular pathways such as the Krebs cycle, the mitochondrial respiratory chain (RC), or in DNA replication and repair [1]. The mitochondrion plays an essential role in the biogenesis of Fe-S. However, the functioning of this essential organelle for cellular energy production is itself dependent on Fe-S cluster-containing proteins. In mammals, Fe-S protein maturation occurs via three distinct and organized biosynthetic pathways. Fe-S cluster biosynthesis always begins in the mitochondrion with de novo [2Fe-2S] cluster formation on the cluster scaffold protein ISCU. The cluster is then transferred to the cluster delivery protein, Glutaredoxin 5 or GLRX5. Maturation of the mitochondrial [2Fe-2S]-containing proteins can directly proceed from GLRX5, while the maturation of the [4Fe-4S]-containing proteins requires supplementary steps with the biosynthesis of the [4Fe-4S] cluster and its delivery to mitochondrial [4Fe-4S] proteins [2,3]. These last steps involve six proteins, namely NUBPL (formerly named IND1), NFU1, BOLA3, IBA57, ISCA2, and ISCA1, whose functions are poorly known and are currently the subject of intense research [4,5] (Figure 1).

First identified in 2011, deleterious variants in the genes encoding for five of these proteins, NFU1, BOLA3, IBA57, ISCA2, and ISCA1, lead to a disease named multiple mitochondrial dysfunction syndrome (MMDS) types 1 to 5, respectively, with symptoms indicative of a general decreased energy metabolism and mitochondrial respiration failure. To date, less than 150 patients with MMDS were reported worldwide. The signs and symptoms of these severe conditions begin early in life, and affected individuals usually do not live past infancy. The most affected tissues in these syndromes are those requiring a lot of energy such as heart, brain, and muscle. Pathophysiology is related to a specific impairment of the maturation of downstream mitochondrial [4Fe-4S]-cluster-containing proteins, which results in a deficiency of mitochondrial respiratory complexes and the impaired function of lipoic-acid-dependent enzymes.

The present review will focus on the MMDS. The methodology we have adopted was based on Pubmed searches using “multiple mitochondrial dysfunction syndrome”, “NFU1”, “BOLA3”, “IBA57”, “ISCA2”, and “ISCA1” as keywords. We included all publications describing a new patient with confirmed genetic analyses.

## 2. Fe-S Proteins: Essential Proteins with Elaborated Maturation Pathways

Since their discovery in bacteria in the 1960s, Fe-S proteins were found to execute a large range of functions in prokaryotic archaea and bacteria as well as in eukaryotic fungi, plants, and animals. They are among the most ubiquitous and versatile metal-containing prosthetic centers and sustain fundamental processes including mitochondrial respiration, the Krebs cycle, DNA metabolism, and cellular iron homeostasis [1,6,7,8,9]. We will start this review by highlighting the essential roles of Fe-S proteins in human cells and their biogenesis pathways.

Fe-S clusters are inorganic prosthetic groups composed of one or more iron ions that are partially or completely coordinated by sulfur atoms. In many proteins, they are associated with other types of cofactors such as organic groups (e.g., flavin) or other metal atoms. Fe-S clusters found in proteins have different atom compositions, cluster structures, and core oxidation states. The most common configurations are the [2Fe-2S], and the [4Fe-4S] core units. The tetrahedral coordination of each iron site is performed by cluster inorganic sulfides (S^2−^) and protein-based ligands, which are generally thiolate groups provided by cysteine residues [1,6,7].

Fe-S clusters are typically redox active. In other words, they can be found in different oxidation states. Thus, each iron ion of the cluster can be either in the oxidized state, Fe(III) (ferric ion), or in the reduced state, Fe(II) (ferrous ion). Fe-S proteins use this property to mediate electron transport (coupled or not to proton transfer) as is the case in the respiratory chain. However, Fe-S clusters can also constitute the active site of a wide range of enzymes including in the [4Fe-4S]-containing aconitase [10] and the members of the fast-growing and diverse radical-SAM (S-adenosyl-L-methionine) superfamily [11]. Fe-S proteins have many other functions such as Fe-S cluster biogenesis (ISCU, ISCA…) or repair (mitoNEET) [12], ribosome synthesis (ABCE1) [13], DNA polymerization and repair [14], sulfur donation (biotin synthase) [15], and the ability to sense different types of environmental stimuli and stress [16,17]. Thus, Fe-S clusters are essential cofactors for various proteins involved in key cellular functions.

The first studies on the biogenesis of Fe-S clusters were performed on bacteria [18] and homologous proteins were then identified in yeast and humans [19,20]. While in vitro chemical reconstitution of Fe-S clusters can occur spontaneously in the presence of proteins that are able to coordinate an Fe-S cluster, it requires several complex protein machineries in vivo to avoid the toxic effects of free iron and inorganic sulfur. In humans, the maturation of Fe-S proteins is a very complex process involving more than 25 proteins, and there are still many grey areas regarding how these proteins cooperate with each other. We will not attempt to describe the entire biogenesis of Fe-S clusters in detail here (see recent reviews including [2,3,5]), but we will give the reader an overview of this process and of the roles of the proteins involved in MMDS.

There are three machineries that have been clearly identified. The mitochondrial Iron-Sulfur Cluster (ISC) assembly machinery is devoted to the formation of the initial Fe-S cluster and to the maturation of mitochondrial Fe-S proteins. An ATP-binding cassette (ABC)-type export machinery brings an unknown sulfur-containing compound X-S, which could also contain iron, out of the mitochondrion to the cytosol [21,22]. Finally, the Cytosolic Iron-sulfur Assembly (CIA) machinery is devoted to the maturation of non-mitochondrial Fe-S proteins. The maturation of all of the mammalian Fe-S proteins always starts in the mitochondrion regardless of the subcellular localization of the protein to be matured.

The mitochondrial process can itself be subdivided in three main steps. First, an initial [2Fe-2S] ^2+^ cluster is built de novo on the scaffold protein ISCU by assembling two Fe^2+^ ions imported into the mitochondrion by the inner mitochondrial membrane transporters, called mitoferrins [23], and two sulfide ions produced from the desulfuration of the cysteines by the NFS1-ISD11-ACP complex [24,25,26]. This first step also requires electrons provided by the ferredoxin FDX2 [27,28,29] and is regulated by frataxin according to a still debated mechanism [30,31]. The newly made [2Fe-2S]^2+^ is then released from ISCU and transferred to the monothiol GLRX5 with the help of the HSPA9/HSC20 chaperone pair. However, recent studies have proposed that ISCU could transfer its cluster to not only GLRX5 but also to specific mitochondrial [2Fe-2S] proteins containing conserved LYR motifs [32,33]. Finally, the GLRX5-bound cluster can be directly inserted into mitochondrial [2Fe-2S]-containing proteins that are then exported out of the mitochondrion or are converted to a [4Fe-4S] cluster.

The late-acting steps of the mitochondrial pathway responsible for the maturation of mitochondrial [4Fe-4S] clusters are still elusive. Proteins mutated in MMDS, namely ISCA1, ISCA2, IBA57, NFU1, and BOLA3, are all involved with NUBPL in these last steps devoted to the maturation of mitochondrial [4Fe-4S]-containing proteins. NUBPL would be exclusively involved in the maturation of [4Fe-4S]-containing proteins of complex I of the RC [34,35]. However, the exact functions of the five other proteins and how they cooperate are still poorly characterized. On the heterocomplex ISCA1-ISCA2, the reductive fusion of two GLRX5-derived [2Fe-2S]^2+^ clusters leading to the formation of [4Fe-4S]^2+^ after the addition of two electrons provided by FDX2 [36]. Some studies involve IBA57 in this step [36,37,38]; however, its implication in the formation of the [4Fe-4S] cluster is still debated [39,40], with some other studies involving IBA57 in the maturation of specific mitochondrial [2Fe-2S] proteins [41]. This [4Fe-4S] cluster can be directly inserted in several mitochondrial [4Fe-4S]-containing proteins, such as the Krebs enzyme aconitase. However, most of them, including the subunits of the respiratory complexes I and II and the lipoic acid synthase (LIAS) involved in the critical modifications of the pyruvate dehydrogenase (PDH) and of the alpha-ketoglutarate dehydrogenase (αKGDH) complexes, require dedicated late-acting ISC targeting factors including NFU1 and BOLA3 for their maturation [42,43,44,45,46]. Deficiency in one of these proteins that is caused by deleterious variants in the corresponding gene leading to multiple mitochondrial [4Fe-4S] protein dysfunction, except for NUBPL, whose deficiency is limited to complex I impairment.

## 3. MMDS Type 1 (MMDS1), Mutation in *NFU1*

Since the first patients described in 2011 [47,48], more than 35 patients with MMDS1 (OMIM#605711) have been reported from all continents (Table 1). This disorder is caused by biallelic pathogenic variants in the *NFU1* gene. Severe encephalopathy was the clinical feature with early onset (less than one year of age) in the initial patients. Their atypical metabolic profiles revealed decreased activity of multiple mitochondrial proteins (PDH, Glycine Cleavage System (GCS), and respiratory chain complexes (RCC)) leading to the first MMDS description.

**Figure 2:**
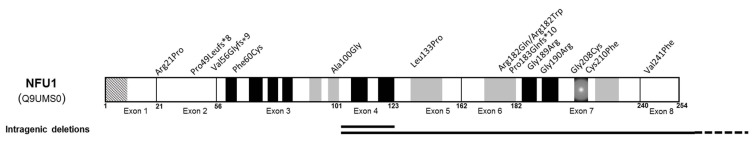
Schematic of NFU1 protein (NP_001002755) with reported variants. Missense and truncating variants are indicated above and intragenic deletions below the protein structure. Schematic secondary structure shows α-helix (grey), β-strand (black), and turn (grey and black crosshatchings). The mitochondrial targeted sequence corresponds to the 10 first residues.

Supplementary analysis on tissues from patients with MMDS1 found that PDH and GCS impairment was secondary to lipoic acid synthesis disruption. These biochemical characteristics suggested that human NFU1 was involved in the maturation of Fe-S cluster-containing proteins, as is the case in its orthologue protein Nfu in plants [61] and in bacteria [62]. Clinical features showed unspecific signs of energetic metabolism disease (axial hypotonia, apnea, myoclonus or seizures, developmental delay) with dramatic evolution. Pulmonary arterial hypertension (PAHT) was reported in several patients and was then linked to a specific genetic variant, which is also the the most frequent, the Gly208Cys, at either the heterozygous or the homozygous state. This variant modifies the native CysXXCys scheme essential for Fe-S carriage by the NFU1 protein to CysXCysXXCys [63]. In vitro analysis revealed functional impairment of NFU1 ^Gly208Cys^ compared to the native protein. The consequence would be an increased retention of the Fe-S cluster by the mutated protein, inhibiting its transfer to acceptor proteins [48,63]. Recently, a study in rats showed that PAHT as well as mitochondrial dysfunction (complexes I and II, and PDH activities) were clearly linked to this variant in *NFU1* [64] with a more drastic effect in homozygous female rats. However, this dimorphism gender was not evident in patients with MMDS1. Hypertrophy in the cardiac left ventricle was not detected in animals carrying this variant. Biochemical analysis showed atypical results for several NFU1 patients compared to other subjects. PDH and RCC activities were indeed not decreased in one patient carrying variant c.545 + 5G > A [54]. Lipoic acid synthesis was not impaired in the fibroblasts of a patient carrying the variant c.179T > C at the homozygous state [50]. These two genotypes could be associated to specific biochemical phenotypes. Among the 13 other variants identified in patients with MMDS1 (Table 1, Figure 2), the second most frequent variant was the missense variant Gly189Arg, which was carried by 5 patients. Some authors have suggested that this variant could be associated with a milder progressive disease [53]. One patient, who was carrying it at the homozygous state, had a later disease onset than usually described and was still alive at 41 months [58]. An Italian patient, a composite heterozygote carrier of the Gly189Arg and Pro49Leufs*8 variants, was the only one to be alive as an adult [52]. His clinical phenotype is clearly atypical compared to that of other patients, as the patient experienced a neurological regression episode at late onset (18 months) with partial recovery. No other decompensation episodes were noticed, and first clinical and biological investigations were only performed at 18 years of age. The predominant clinical feature at adult stage was described as peripheral neuropathy mimicking PDH deficiency, the activity of which was deeply decreased in the fibroblasts. Other classical biochemical findings in NFU1 patients (high glycine levels, decreased RC activities) were also present. Indeed, except for these two patients, all of the others died in the first three years of life regardless of the genotype. Neither treatment based on lipoic acid or other vitamin cocktails nor a ketogenic diet was efficient [49]. The authors suggested epigenetic factors as possible modulator effects and as an explanation for the exceptional life span of the Italian patient [52]. Metabolomic investigations in different tissues or body fluids could be helpful to provide further compensatory pathways involved in several tissues of this patient.

## 4. MMDS Type 2, Mutations in *BOLA3*

Variants in *BOLA3* cause MMDS type 2 (OMIM#614299), which can be described as a severe mitochondrial disease leading to early death in almost all patients. The first patient was reported 10 years ago [47] and the clinical phenotype showed severe encephalopathy with respiratory distress, seizures, failure to thrive, and dilated cardiomyopathy. Biochemical findings in this patient resembled those of MMDS1, which included increased glycine levels in body fluids and decreased activity of complexes I and II of the RC and PDH. The lipoylation of mitochondrial proteins was also impaired. Since 2011, at least fifteen patients from worldwide origins were reported (Table 2).

The clinical phenotype was similar to that of the first MMDS2 patient, and cardiomyopathy was mentioned in half of the patients. Hypertrophic and dilated cardiomyopathies were described. Psychomotor regression was frequently the first symptom reported and began in the first year of life in most of the patients. Optic atrophy or nystagmus were reported in three patients [66,70]. In one patient, acute right hemiparesis revealed the disease at 18 months of age [72].

Brain imaging findings showed bilateral periventricular and deep white matter until spinal cord lesions formed in most cases, without further specification. Demyelination was also frequently observed. Biochemical analyses were performed using different tissues of several patients. As expected, high levels of glycine and lactate in body fluids and decreased PDH and complex I and II activity were found in most patients. However, the activities of these complexes were normal for a few patients. Thus, MMDS2 could not be ruled out for a patient with normal RCC activity.

**Figure 3:**
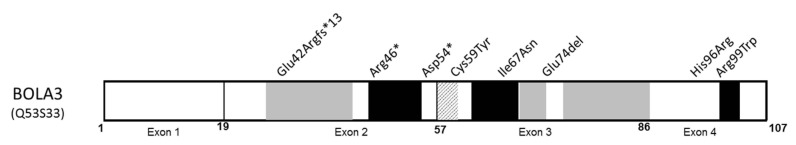
Schematic of BOLA3 protein (NP_997717) with reported variants. Missense variants are indicated in italic. Schematic secondary structure shows α-helix (grey), β-strand (black), and turn (grey and white crosshatchings) (adapted from UniprotKB, protein Q53S33). A mitochondrial targeted sequence has not yet been formally identified.

Among the 18 reported patients from 14 families worldwide, only 8 variants were identified (Figure 3).

The three types are either stop, frameshift mutation, or deletion in the second exon, while the missense mutations are localized in exons 3 and 4. The mutations Cys59Tyr and His96Arg affect highly conserved residues that are crucial for protein functionality [74]. Other missense variants modified amino acids involved in the β-strands. His96Arg was only identified in the Japanese patients. It is noteworthy that both patients carrying the homozygous variant Arg99Trp presented a milder phenotype than the others [69]. Of those two patients, one was the only patient still alive at 12 years of age, and no cardiomyopathy was mentioned in his clinical description. The second one could have benefitted from immunomodulation treatment (methylprednisolone), which could have led to partial improvement without long-term follow-up for this patient. The other reported patients were often treated with classical vitamin cocktails without clear effectiveness [75]. A milder clinical phenotype with longer survival was observed in patients carrying the Cys59Tyr variant, which is also [72] due to the partial dysfunction of mutated protein [76].

BOLA3 is indeed involved in the delivery of an [4Fe-4S] cluster from GLRX5 to recipient mitochondrial Fe-S proteins with the help of NFU1. Both NFU1 and BOLA3 deficiencies lead to similar clinical phenotypes, including severe encephalopathy and cardiac impairment. In MMDS1, pulmonary hypertension is clearly related to one pathogenic variant (Gly208Cys) without hypertrophic cardiomyopathy for most patients. However, to date, no animal experiments have been performed to improve knowledge regarding the cardiomyopathy detected in MMDS2 patients. Recently, in vitro assessment of BOLA3 knockdown in human pulmonary artery endothelial cells confirmed that BOLA3 regulates glycolysis, mitochondrial Fe-S protein maturation, and mitochondrial respiration activity [77]. In this human cellular model, BOLA3 deficiency leads to increased endothelial vasoconstriction, while in mice cells, it causes histological modifications typical of those found in pulmonary hypertension.

## 5. MMDS Type 3 (MMDS3), Mutations in *IBA57*

Biallelic pathogenic variants in *IBA57* leading to MMDS type 3 were first reported in a family involving two siblings [78]. They presented with antenatal signs of polyhydramnios and microcephaly. Biochemical studies in fibroblasts and muscle identified decreased activity in the RC complexes I, II, and IV and in the lipoylated proteins. Both patients died in the neonatal period. Since this first publication, 25 other families with MMDS3 involving 57 patients have been reported without additional patients with antenatal signs (Table 3).

**Figure 4:**
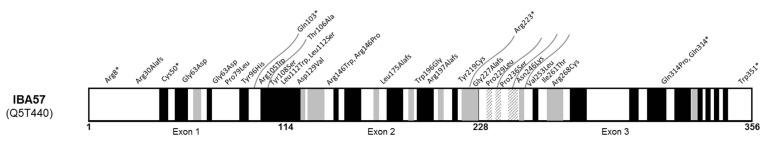
Schematic of IBA57 protein (NP_001010867) with reported variants. Schematic secondary structure shows α-helix (grey), β-strand (black), and turn (grey and white crosshatches) (adapted from UniprotKB, protein Q5T440).

Eye findings such as optic atrophy and/or nystagmus or abnormal vision evoked potentials were present in almost half of the patients (28/59). Psychomotor regression with progressive spasticity potentially leading to quadriparesis was the most frequent initial clinical feature with variable onset age. However, more than 20 patients were still alive after 5 years of age. For one patient, treatment with riboflavin, coenzyme Q10, and baclofene led to a slow clinical recovery, and seizures were efficiently treated by levetiracetam [75]. Brain imaging of the described patients revealed very frequent cavitating leukoencephalopathy (Table 3). Interestingly, in a cohort of 37 patients with this imaging feature, 46% were carrying IBA57 variants [84]. Biochemical findings were hyperlactatemia in most of the patients. A high level of blood glycine was found in only a few patients. The activity of the RC complexes I, II, and IV were often impaired in patients who were also associated with a low level of lipoylated proteins in the fibroblasts. Nevertheless, RC was not systematically impaired in the fibroblasts [75,82]. To date, 34 pathogenic variants in IBA57 have been identified without frequent variant nor evident genotype- phenotype correlation (Figure 4). However, the variant c.678A > G identified in 12 related patients of Palestinian origin could be associated with the SPOAN-like phenotype (spastic paraplegia, optic atrophy and peripheral neuropathy) [79]. All of these patients survived until adulthood. Biochemical findings in the fibroblasts showed residual lipoylation of mitochondrial proteins suggesting the residual efficiency of mutated IBA57 in the Fe-S protein maturation pathway resulting in a milder clinical phenotype.

## 6. MMDS Type 4, Mutations in *ISCA2*

In 2015, seven patients from six families exhibiting severe neurodevelopmental regression, spasticity, and optic atrophy were reported [86,87]. Molecular studies revealed a homozygous variant in *ISCA2* for each patient, the Gly77Ser variant, leading to the description of the first patients with MMDS 4 (OMIM#616370). To date 24 patients have been reported, including 19 Saudi patients carrying the same variant [88,89,90] (Table 4).

Clinical and neuroimaging findings were quite similar between all of the described patients. Initial psychomotor development was normal and clinical status was deteriorated during the first year of life leading to spasticity and visual impairment (nystagmus and/or optic atrophy), as is the case for other mitochondrial disorders. Lesions in cerebral and cerebellar white matter and the spinal cord were indeed quite constantly present and may suggest a leukoencephalopathy with brainstem and spinal cord involvement and high brain lactate (LBSL) caused by the *DARS2* mutation [91]. Death could occur in the neonatal period or in the first years of life (Table 4). Most of the surviving adolescent patients exhibited an extremely severe encephalopathy. To date, no patient has lived to adulthood.

**Figure 5:**
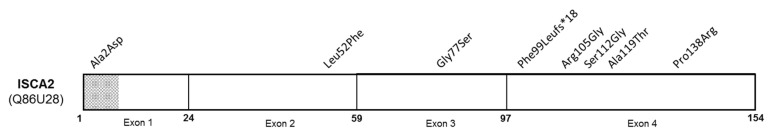
Schematic of ISCA2 protein (NP_919255) with reported pathogenic variants. Mitochondrial target sequencing encompassed residues 1 to 8 (grey and white crosshatches) (adapted from UniprotKB, protein Q86U28).

Biochemical analyses including an RC functional study showed that all complexes might be affected [93] but the activity of complexes III and V were rarely decreased in the tissues of patients with ISCA2 deficiency [50,89]. Lipoylated proteins (PDH, KGDH) were also decreased in the fibroblasts of patients, in accordance with the involvement of ISCA2 in the maturation of mitochondrial [4Fe-4S] proteins [94]. Interestingly, mitochondrial morphology with mitochondrial network disruption was abnormal in the fibroblasts of ISCA2 deficient patients [89]. Mitochondrial DNA depletion was also present in several studies, including in fibroblasts from Saudi ISCA2 deficient patients [86,89]. This variant seemed to be a founder mutation in Saudi families, while in five other patients from different countries, seven sporadic variants were identified. These seven variants affect all regions of the ISCA2 protein (Figure 5).

## 7. MMDS Type 5, Mutations in *ISCA1*

Pathogenic variants in *ISCA1* lead to MMDS type 5, the most recently described MMDS [95]. The first four reported cases exhibited severe neonatal encephalopathy. Among the seven described cases from five families, five were from Indian families [95,96], while the other patients were of Italian [97] and Egyptian [98] origins. All of the Indian patients carried the same homozygous variant Gln87Lys, whereas the homozygous variants Val10Gly and Tyr101Cys were identified in the Italian and Egyptian patients, respectively (Table 5). Biochemical investigations showed expected hyperlactatemia and elevated urinary glycine level (one case). Elevated creatine phosphokinase (CPK) was reported in one of the two tested Indian patients. Mitochondrial protein studies were performed in tissues of patients carrying the Val10Gly and Tyr101Cys variants. The lipoylation of the mitochondrial proteins was impaired. The functionality of the RCC was differently affected depending on the analyzed tissues, but the dysfunction of complexes II and IV was always observed [97,98]. These findings suggest that ISCA1 impairment does not affect the maturation of [2Fe-2S] proteins (including the Rieske protein of complex III but only that of the mitochondrial [4Fe-4S] proteins [97].

From all the seven ISCA1 cases, two clinical presentations would be summarized as (i) severe early encephalopathy with major psychomotor retardation and few or no achieved milestones leading to early death or (ii) moderate encephalopathy. The first category is linked to Indian patient genotype while the second category is linked to the others. Spasticity (*n* = 7/7), nystagmus (*n* = 2/7), and seizures (*n* = 2/7) were the other neurological symptoms. However, there was an early mortality for most of the patients: among seven cases, five of them died between 11 months and 11 years old.

Brain magnetic resonance imaging (MRI) showed frequent abnormalities such as ventriculomegaly, but other imaging data were associated with different genotypes. Cerebral and cerebellar white matter leukodystrophy and pachygyria were present in patients carrying the Gln87Lys variant, while thin corpus callosum associated with vacuolating leukodystrophy were described in other patients. As expected, the lactate peak in brain magnetic resonance (MR) spectroscopy was also present in the majority of affected individuals.

To date, the seven reported patients with MMDS type 5 presented some similar clinical features as individuals with mitochondrial cytopathy or PDH deficiency without distinctive imaging or a biochemical phenotype compared to other MMDS.

## 8. Conclusions

Since the development of next generation sequencing and whole exome (or genome) sequencing in the year 2010, pathogenic variants in genes encoding for the proteins involved in the late steps of the mitochondrial Fe-S protein maturation pathway were identified in diseased patients. They exhibited severe or fatal early onset encephalopathy with multiple biochemical abnormalities (RCC, PDH, Krebs enzymes impairment) and secondary to mitochondrial [4Fe-4S] proteins maturation disorder. Despite classification of MMDS into five different categories according to the mutated gene, clinical symptoms, brain imaging, and biochemical components, findings are quite homogeneous, resembling other severe mitochondrial leukoencephalopathies such as LBSL, for instance. A lactate peak on MR spectroscopy was also often observed as in classic mitochondriopathies. The ophthalmologic manifestations usually present in patients with other mitochondrial diseases [99] and have frequently been reported in MMDS types 3, 4, and 5; rarely in MMDS type 2; and never in type 1. The reason why these manifestations are inconstant in patients with MMDS remains unknown, but it suggests that an ophthalmological examination is needed for all patients with established MMDS. Associated with encephalopathy, cardiac impairment such as PAHT (isolated or not) and dilated cardiomyopathy could suggest MMDS type 1 or type 2, respectively. With respect to specific biochemical findings, the accumulation of glycine and lactate in body fluids was frequently, albeit not always, noted as were decreased RCC activities.

## Figures and Tables

**Table 1:** Reported patients with MMDS type 1.

Ref.	Nb of Patients (Nb and Origin of Families)	Age at Onset/Age at Death	Metabolic Findings	Impaired Mitochondrial Proteins	Pulmonary Hypertension	Clinical Feature	Brain MRI/ Spectroscopy	Genotype (*NFU1*:NM_001002755.4)
[47]	3 (1F, Mexico)	Neonatal/1st m	High lactate and glycine levels (bl)	Complexes I + III, II, IV, PDH, KGDH, lipoylated proteins	No	FD, hypotonia, respiratory failure	NA	c.[545G > A]; [545G > A]; p.[(Arg182Gln)]; [(Arg182Gln)]
[48]	10 (9F incl. 4F of basque origin)	1–9 m/2–15 m	High lactate and glycine (bl and CSF) and amino-adipate, α-KG, 2 ketoadipate, tyglyglycine (ur) levels	PDH, lipoylated proteins	Yes (7/10)	FD (6/10), dysmorphia (1/10), skills regression (6/10)	LD and semioval necrosis (*n* = 1), semioval center and cerebellum lesions (*n* = 1), cerebral atrophy (*n* = 1)	c.[622G > T]; [622G > T], p.[(Gly208Cys)]; [(Gly208Cys)] (9/10) c.[622G > T]; [545 + 5G > A], p.[(Gly208Cys)]; [Pro183Glnfs*10] (1/10)
[49,50]	1 (France)	5 m/3 y	High lactate (bl), glycine (bl and CSF) and α-KG (ur) levels	Complexes I, II, PDH, KGDH, lipoylated proteins	In acute episodes	Severe DR, transient MD, myoclonies, hypotonia	Progressive LE, kysts in PV WM and in CC	c.[565G > A]; [622G > T], p.[(Gly189Arg)]; [(Gly208Cys)]
[51]	1 (Italia)	7 m/18 m	High lactate, pyruvate, glycine (bl), succinate, fumarate, glutarate (ur) levels	Complexes I, II, IV, PDH	No	FD, spasticity, severe DR	Diffuse LE (deep WM), cavitations/peak of lactate	c.[565G > A]; [629G > T], p.[(Gly189Arg)]; [(Cys210Phe)]
[52]	1 (unknown)	18 m/alive at 30 y	High lactate, pyruvate (bl), glycine (bl and CSF) levels	Complexes II, IV, PDH, KGDH,	No	Spastic tetraparesis, scoliosis, DR after fever, MSN	PV WM and CC abnormal signal, CC atrophy/reduced NAA peak, mild lactate, and high choline peaks	c.[146del]; [565G > A], p.[(Pro49Leufs*8)]; [(Gly189Arg)]
[53]	7 (5F, Germany (*G*), Romania/Serbia (*RS*) Pakistan (*P*), Belgium (*B*))	Birth–5 m/3 m–30 m	High lactate (6/7), glycine (7/7) levels (bl)	Complexes I (2/5), II (3/3), IV (1/4), PDH (5/5)	Yes (4/7)	Muscular hypotonia (6/7), apnea (4/7), FD (4/7), tubulopathy (1/7), CMP (2/7)	LE (3/4) affecting PV (2/4), capsula interna (3/4), or upper cervical cord (1/4); hypomyelination (2/4)	g.[69400462C > A]; [69592691_69648327del], p.[(Gly208Cys)]; [?] (G) c.[565G > A]; [568G > A], p.[(Gly189Arg)]; [(Gly190Arg)] (G) c.[544C > T]; [?], p.[(Arg182Trp)]; [?]) (RS) c.[302 + 3A > G]; [302 + 3A > G], p.[(Val56Glyfs∗9)]; [(Val56Glyfs∗9)] (3/7, P) c.[62G > C]; [622G > T], p.[(Arg21Pro)]; [(Gly208Cys)] (B)
[54]	2 (2F, unknown)	2–15 m/14–19 m	High lactate (CSF) and glycine (ur, bl and CSF), 2-amino and 2-hydroxyadipate, tyglylglycine, isovalerylglycine (ur) levels	GCS (1/1), lipoylated proteins (2/2), CII + CIII (1/1)	Yes (1/2)	Severe spasticity, axial hypotonia, apnea, DR	cysts in temporal lobes with glycine peak (1/2), WM spongiosis, PV WM and CC LD (1/2)	c.[622G> T]; [545 + 5G > A], p.[(Gly208Cys)]; [Pro183Glnfs*10]
[55]	2 (2F, China)	3–4 m/3–4 m	High lactate level (bl, ur, 1/2), normal glycine levels (2/2)	NA	Yes (1/2)	DR (2/2), seizures, muscular hypertonia, and ascitis (1/2)	LD in subcortical WM (internal—external capsule, CC, medulla) or midbrain, thalamus, lateral ventricle, and centrum ovale	c.[545 + 5G > A]; [545 + 5G > A], p.[(Pro183Glnfs*10)]; [(Pro183Glnfs*10)] c.[721G > T]; [303_369del], p.[(Val241Phe)]; [?]
[50]	3 (3F, unknown)	3–3, 5–7 m/4 m to 3 y	High lactate (bl 3/3) and glycine (bl:2/2, ur:1/2 and CSF:1/1), fumarate (1/1), 2-amino-adipate (pl) or 2 keto-adipate (ur) levels	Complexes I (3/3), II (2/3), IV (1/3), PDH (3/3), KGDH (2/3), lipoylated proteins (2/3)	Yes (1/3)	Seizures (2/3), spasticity (1/3), apnea (1/3), PMD (2/3)	Normal (1/3), WM abnormalities (1/3), necrotizing LD (1/3) /lactate peak (1/2)	c.[146del], [303–1988_c.369 + 1021del], p.[(Pro49Leufs*8)]; [?] c.[622G > T]; [629G > T], p.[(Gly208Cys)]; [(Cys210Phe)] c.[179T > G]; [179T > G], p.[(Phe60Cys)]; [(Phe60Cys)]
[56]	1 (Brazil)	4 m/8 m	High lactate (bl, ur) and glutaric acid (ur) levels	NA	No (normal ECG)	Severe hypotonia, spastic tetraparesis, DR, lethargy	Cavitating cystic LE (PV, deep, subcortical areas)	c.[545G > A]; [622G > T], p.[(Arg182Gln)]; [(Gly208Cys)]
[57]	1 (Portugal)	1 m/10 m	Elevated glycine level (bl)	NA	Severe (onset)	FD, DR, seizures, spasticity, and axial hypotonia	WM necrotic areas, PV WM abnormal signals	c.[622G > T]; [622G > T], p.[(Gly208Cys)]; [(Gly208Cys)]
[58]	1 (Turkey)	23 m/alive at 41 m	High lactate and glycine (bl) levels	NA	No (normal ECG)	Episodes of DR and legs spasticity after fever	Frontal PV cysts and frontal WM and CC hyperintensity,	c.[565G > A]; [565G > A], p.[(Gly189Arg)]; [(Gly189Arg)]
[59]	2 (1F, Guatemala)	2–3 m/5–7 m	High lactate (bl) and α-KG (ur) levels	E3 and PDH	Yes (with cardiomegaly or dilated CMP)	FD, emesis, developmental delay, spasticity	NA	c.[544C > T]; [622G > T], p.[(Arg182Trp)]; [(Gly208Cys)]
[60]	1 (unknown)	7 m/12 m	High glycine (bl, CSF) and lactate (CSF) levels	NA	Yes (mild)	PMD, bradypnea, hypotonia	Bilateral basal ganglia, thalami, frontoparietal WM, cerebral peduncles, spinal cord abnormal signals/lactate peak (MRS)	c.[299C > G]; [398T > C], p.[(Ala100Gly)]; [(Leu133Pro)]

Bl: blood; CC: corpus callosum; CMP: cardiomyopathy; CSF: cerebrospinal fluid; DR: developmental regression; ECG: electrocardiogram; F: families; FD: feeding difficulties; GCS: glycine cleavage system; α-KG: alpha-ketoglutarate; LD: leukodystrophy; LE: leukoencephalopathy; m: month(s); MD: motor deficiency; MRI: Magnetic resonance imaging; MRS: Magnetic resonance spectroscopy; MSN: motor-sensory neuropathy; NA: not available; NAA: N-acetylaspartate; PMD: psychomotor delay; PV: periventricular; ur: urine; WM: white matter; y: year(s).

**Table 2:** Reported patients with MMDS type 2.

Ref.	Nb of Patients (Nb and Origin of Families)	Age at Onset/Age at Death	Metabolic Findings	Impaired Mitochondrial Proteins	Cardiomyo- pathy	Clinical Feature	Brain MRI/Spectroscopy	Genotype (*BOLA3*:NM_212552.2)
[47]	1 (East Indian)	4 m/11 m	High glycine level (bl, ur)	Complexes I and II, PDH, lipoylated protein	Dilated	FD, lethargy, respiratory distress, HMG, DD	NA	c.[123dupA]; [123dupA], p.[(Glu42Argfs*13)]; [(Glu42Argfs*13)]
[65]	2 (1F, unknown)	<1 m/3 m	High pyruvate (bl, ur), lactate (bl, ur), and glycine levels (bl)	Complexes I, II, IV, PDH, lipoylated protein	Hyper-trophic	FD, SE, hypotonia, progressive encephalopathy	Delayed myelination, supra-, PV, and bifrontal and biparietal hyperintense lesions (T2)	c.[200T > A]; [200T > A], p.[(Ile67Asn)]; [(Ile67Asn)]
[66]	3 (3F: Australia, India, African- American)	6–8 m/7 m, 22 m, 11 y	High lactate (bl), glycine (CSF) levels	Complex II (*n* = 1/3), PDH (*n* = 1/2), lipoylated protein (*n* = 2/2), GCS (*n* = 3/3)	Hyper-trophic (*n* = 2/2)	FD, SE (*n* = 1/3), Myoclonus (2/3), SP and severe PMDD (1/3), skills regression (2/3), optic atrophy (*n* = 2/3)	PV WM and spinal cord lesions (*n* = 1/3), subcortical fibers lesions (*n* = 1/3) LD (*n* = 2/3), cerebral and cerebellar atrophy (*n* = 1/3)	c.[136C > T]; [136C > T], p.[(Arg46*)]; [(Arg46*)]
[67,68]	4 (4F, Japan)	<1, 2, 6 m/3–11 m	Metabolic acidosis	Combined complex deficiencies (*n* = 4/4)	Hyper-trophic (*n* = 3/4)	Renal, respiratory, and/or liver failures, SE, FCMD (*n* = 1)	NA	c.[287A > G]; [287A > G], p.[(His96Arg)]; [(His96Arg)] (3/4) c.[225_229del]; [287A > G], p.[(Lys75Asnfs*9)]; [(His96Arg)]
[50]	2 (1F, India)	5–7 m/8 m	High pyruvate (bl), lactate (bl, CSF), glycine (bl, CSF, ur) levels	Complexes I, II, IV, PDH, lipoylated protein	No	FD, tetraparesis, hypotonia, PMDD	Diffuse demyelination/lactate peak	c.[136C > T]; [136C > T], p.[(Arg46*)]; [(Arg46*)]
[69]	2 (unknown)	18 m/alive at 12 y (*n* = 1)	NA	NA	NA	SPT (*n* = 1/2), skills regression, gait difficulty	PV and deep WM or fronto-parietal lesions/lactate peak (*n* = 1/1)	c.[295C > T]; [295C > T], p.[(Arg99Trp)]; [(Arg99Trp)]
[70]	1 (unknown)	8 m/15 m	High lactate (bl), tiglylglycine (ur) levels	PDH, lipoylated protein	NA	FD, hypertonicity then hypotonia, nystagmus	Symmetric ovoid areas and CC, cervical cord regions abnormal signal. PV, deep, subcortical WM lesions/lactate peak	c.[200T > A]; [220_222del], p.[(Ile67Asn)]; [(Glu74del)]
[71]	1 (Japan)	6 m/10 m	High lactate (bl, CSF), glycine (bl) levels	Complexes I, II, IV	Hypertrophic	FD, SE, eyelid movements, hypotonia, skill regression	Deep WM to spinal cord lesions	c.[287A > G]; [287A > G], p.[(His96Arg); [(His96Arg)]
[72]	1 (England)	18 m/alive at 8 y	Normal lactate (bl), high glycine (bl, ur) levels	PDH, normal RCC activity but low quantity of protein in complexes I and II	No	Ataxia, acute hemiparesis, cognitive regression, congenital hypothyroidism	subcortical WM, basal ganglia, brainstem and cerebellum abnormal signal and dysmyelination	c.[136C > T]; [176G > A], p.[(Arg46*)]; [(Cys59Tyr)]
[73]	1 (South Africa)	7 m/17 m	High lactate (bl), glycine (ur) levels	PDH	No	Sudden skill regression, floppiness, encephalopathy	Demyelination	c.[159dupT]; [159dupT], p.[(Asp54*)]; [(Asp54*)]

Bl: blood; CC: corpus callosum; CSF: cerebrospinal fluid; DD: developmental delay; F: families; FCMD: Fukuyama congenital muscular dystrophy; FD: feeding difficulties; HMG: hepatomegaly; m: month(s); MRI: Magnetic resonance imaging; NA: not available; PDH: pyruvate dehydrogenase complex; PMDD: psycho-motor developmental delay; PV: periventricular; RCC: respiratory chain complexes; SE: seizures; SP: spasticity; SPT: spastic tetraparesis; Ur: urine; WM: white matter; y: year(s).

**Table 3:** Reported cases with MMDS type 3.

Ref.	Nb of Patients (Nb and Origin of Families)	Age at Onset/Age at Death	Metabolic Findings	Impaired Mitochondrial Proteins	Ophthalmological Findings	Clinical Feature	Brain MRI/Spectroscopy	Genotype (*IBA57*: NM_001010867)
[78]	2 (1F, Morocco)	Antenatal/1 d, 15 d	Increased lactate (bl) and glycine (bl, CSF) levels	Complexes I, II, and IV, lipoylated proteins, IBA57 protein	NA	Polyhydramnios, microcephaly, severe hypotonia	Cerebral atrophy, polymicrogyria, CC hypoplasia	c.[941A > C]; [941A > C], p.[(Gln314Pro)]; [(Gln314Pro)]
[79]	12 (1F, Palestine)	After 3 y/alive (12–65 y)	Normal lactate level	Complexes I, II, lipoylated proteins	Optic atrophy	Peripheral neuropathy, progressive spastic paraplegia	Hyperintensity in WM, cystic cavitation. Cerebellar, CC, CSC atrophy	c.[678A > G]; [678A > G], p.[Gly227Valfs]; [Gly227Valfs]
[80]	1 (Morocco)	6 m/17 m	Increased lactate and glycine levels (bl, CSF)	Complexes I, II, and IV, lipoylated proteins,	NA	FD with vomiting, opistothonos crises, progressive hypotonia, skills regression	Central and PV LE involving CC IC, USC, and cerebellar WM/lactate peak	c.[436C > T]; [436C > T], p.[(Arg146Trp)]; [(Arg146Trp)]
[50]	2 (2F, unknown)	3 w–8 m/2–11 m	High lactate level (bl, CSF)	Complexes I, II, lipoylated proteins, PDHc	NA	Hypotonia, with recurrent myoclonus, pyramidal syndrome, apnea	PV LD, myelination delay	c.[335T > G]; [437G > C], p.[(Leu112Trp)]; [(Arg146Pro)] c.[316A > G]; [738C > G]; p.[(Thr106Ala)]; [(Asn246Lys)]
[81]	3 (2F, unknown)	11 m–2 y/alive at 6–7 y	Intermittent high lactate level (bl, CSF)	Complexes I, II, lipoylated proteins, IBA57 protein level	NA	Spasticity, axial hypotonia, PMR	PV and CC LE with cysts or cavitation	c.[323A > C]; [940C > T], p.[(Tyr108Ser)]; [(Gln314*)] c.[323A > C]; [150C > A], p.[(Tyr108Ser)]; [(Cys50*)]
[75]	4 (4F, Tunisia, unknown)	4–18 m/2 y (*n* = 2) or alive at 12–16 y	High lactate and pyruvate levels (bl or CSF, 3/4)	Complex II (fib: 3/4, muscle 2/3)), complex I fib (1/4)	Nystagmus (2/4), abnormal VEP (3/3)	Severe PMR (4/4), FD (3/4), spastic tetraparesis (3/4), severe hypotonia (1/4), epilepsy (1/4)	Cavitating LE or LD (3/4), cerebral and cerebellar WM involvement (3/4)/lactate peak (3/4)	c.[586T > G]; [c.686C > T], p.[(Trp196Gly]; [Pro229Leu] c.[87insGCCCAAGGTGC]; [313C > T], p.[(Arg30Alafs)]; [(Arg105Trp)] c.[706C > T]; [706C > T], p.[(Pro236Ser)]; [(Pro236Ser)] c.[316A > G]; [757G > C]; p.[(Thr106Ala]; [(Val253Leu)]
[69]	2 (2F, unknown))	18–20 m/alive at 19–31 m	NA	NA	NA	Milestones loss, difficulty in standing, progressive quadriparesis	Large hyperintense lesions in FP WM (2/2), in CC, and multiple WM cysts (1/2)	c.[738C > G]; [802C > T], p.[(Asn246Lys)]; [(Arg268Cys)] c.[656A > G]; [706C > T], p.[(Tyr219Cys)]; [(Pro236Ser)]
[82]	3 (2F, Israel, Japan)	7–20 m/alive at 7–19–29 y	Normal lactate level (1/1)	Normal RCC activity (1/1)	Reduced vision with pale discs (2/3), pendular nystagmus (1/3); no (1/3)	No neurological signs (1/3), spasticity with learning disabilities (1/3), PMR, spasticity, seizures, severe hypotonia and PMD (1/3)	PV WM hypersignal, thin optic nerves, PV rarefaction with thin CC (2/3), progressive WM atrophy, and cystic degeneration (1/3)	c.[335T > C]; [c.588dup], p.[(Leu112Ser)]; [(Arg197Alafs)] c.[386A > T]; [731A > C], p.[(Asp129Val)]; [(Glu244Ala)]
[83]	11 (9F, China)	5–15 m/Alive at 11 m to 10 y	High blood lactate level (2/11)	NA	Nystagmus (3/11), visual impairment 3/11	Spasticity and motor regression (11/11), seizures (2/11)	Cavitating lesions in the frontal, parieto-occipital WM, CC (7/11), temporal (4/11), cerebellar, or IC (1/11)	c.[286T > C]; [316A > G], p.[(Tyr96His)]; [(Thr106Ala)] (*n* = 2) c.[701A > G]; [782T > C], p.[(Asp234Gly)]; [(Ile261Thr)] c.[286T > C]; [697C > T], p.[(Tyr96His)]; [(Arg223*)] (*n* = 3) c.[188G > A]; [286T > C], p.[(Gly63Asp)]; [(Tyr96His)] c.[286T > C]; [307C > T], p.[(Tyr96His)]; [(Gln103*)] (*n* = 2) c.[286T > C]; [754G > T], p.[(Tyr96His)]; [(Gly252Cys)] c.[22C > T]; [286T > C], p.[(Arg8*)]; [(Tyr96His)]
[84]	17 (unknown, China)	NA	NA	NA	Visual impairment (8/17)	NA	No progressive cavitating LE: involving diffuse (12/17 or deep WM (5/17),	c.[22C > T]; [286T > C], p.[(Arg8*)]; [(Tyr96His)] c.[188G > A]; [286T > C], p.[(Gly63Asp)]; [(Tyr96His)] (*n* = 2) c.[236C > T]; [307C > T], p.[(Pro79Leu)]; [(Gln103*)] c.[286T > C]; [697C > T], p.[(Tyr96His)]; [(Arg223*)] (*n* = 4) c.[286T > C]; [307C > T], p.[(Tyr96His)]; [(Gln103*)] (*n* = 2) c.[286T > C]; [316A > G], p.[(Tyr96His)]; [(Thr106Ala)] (*n* = 2) c.[286T > C]; [522_523del], p.[(Tyr96His)]; [(Leu175Alafs)] c.[286T > C]; [589_590del], p.[(Tyr96His)]; [(Arg197Alafs)] c.[286T > C]; [754G > T], p.[(Tyr96His)]; [(Gly252Cys)] c.[286T > C]; [1053G > A], p.[(Tyr96His)]; [(Trp351*)] c.[701A > G]; [782T > C], p.[(Asp234Gly)]; [(Ile261Thr)]
[85]	2 (2F, China)	5–15 m/unknown	Normal lactate level	NA	NA	PMR	LE	c.[286T > C]; [697C > T], p.[(Tyr96His)]; [(Arg223*)] c.[286T > C]; [589_590del], p.[(Tyr96His)]; [(Arg197Alafs)]

Bl: blood; CC: corpus callosum; CSC: cervical spinal cord; CSF: cerebrospinal fluid; d: day(s); F: families; FD: feeding difficulties; fib: fibroblast; FP: frontoparietal; IC: internal capsule; LD/LE: leuko-dystrophy/encephalopathy; m: month(s); MRI: Magnetic resonance imaging; NA: not available; PMR/D: psychomotor regression/delay; PV: periventricular; RCC: respiratory chain complexes; USC: upper spinal cord; VEP: visual evoked potentials; w: week(s); WM: white matter; y: year(s).

**Table 4:** Reported cases with MMDS type 4.

Ref.	Nb of Patients (Nb and Origin of Families)	Age at Onset/Age at Death	Metabolic Findings	Impaired Mitochondrial Proteins	Ophthalmological Findings	Clinical Feature	Brain MRI/Spectroscopy	Genotype (ISCA2: NM_194279)
[86]	6 (5F, Saudi Arabia)	3–7 m/11 m to 5 y (*n* = 4), alive (13–16 m, *n* = 2)	Negative	Complex I	Optic atrophy (6/6)	Spasticity, severe PMR	Lesions in cerebral (PV region to U fibers) and cerebellar WM, CC, internal capsule	c.[229G > A]; [229G > A], p.[(Gly77Ser)]; [(Gly77Ser)]
[87]	1 (Saudi Arabia)	NA	NA	NA	NA	Neurodegeneration	Lesions in WM/Lactate peak	c.[229G > A]; [229G > A], p.[(Gly77Ser)]; [(Gly77Ser)]
[50]	2 (2F, unknown)	2 d, NA/12 d (*n* = 1), alive at 12 y	Elevated lactate (bl, CSF 1/2)	Complexes I, II, lipoylated protein	No	FD, spasticity (1/2), severe hypotonia	Leukodystrophy (1/2) WM atrophy (1/2)	c.[154C > T]; [154C > T], p.[(Leu52Phe)]; [(Leu52Phe)] c.[313A > G]; [313A > G], p.[(Arg105Gly)]; [(Arg105Gly)]
[88]	10 (9F, Saudi Arabia)	3–7 m/11–28 m (*n* = 5), alive between 10 to 34 m (*n* = 5)	Elevated lactate and glycine levels (bl, 2/2)	NA	Optic atrophy and nystagmus	Seizures 2/10, axial hypotonia with peripheral spasticity	Severe PMR/lactate peak (1/2), glycine peak (2/2)	c.[229G > A]; [229G > A], p.[(Gly77Ser)]; [(Gly77Ser)]
[89]	2 (2F, Saudi Arabia)	3–6 m/2 y, alive at 3 y	Elevated lactate (CSF), glycine, and glutamate (CSF, ur) levels	Complexes II, IV, lipoylated proteins, mtDNA depletion	Optic atrophy and nystagmus	PMR (2/2), spasticity, macrocephaly and syndactyly (1/2)	Lesions in cerebral (PV region to U fibers 1/2) and cerebellar WM (2/2), CC, internal capsule, spinal cord (1/2)/lactate peak (2/2)	c.[229G > A]; [229G > A], p.[(Gly77Ser)]; [(Gly77Ser)]
[91]	1 (Italy)	2 m/3 m	Initially normal, elevated lactate level (CSF, later)	Complexes II and IV (muscle)	Nystagmus, normal fundus oculi	Progressive severe hypotonia	Lesions in cerebral grey and WM (U fibers, CC, spinal cord)	c.[295delT]; [334A > G], p.[(Phe99Leufs*18)]; [(Ser112Gly)]
[92]	1 (Iran)	7 m/NA	NA	NA	NA	Hypotonia, irritability, malaise, muscle stiffness	Lesions in PV WM; centrum semiovale, cerebellar peduncles/peak of lactate, increase choline	c.[355G > A]; [355G > A], p.[(Ala119Thr)]; [(Ala119Thr)]
[93]	1 (Yemen/Tunisia)	11 m/alive 11 y	Mildly elevated lactate (bl, CSF)	All respiratory chain complexes	Nystagmus	Severe hypotonia, PMR, spasticity later,	Progressive lesions in PV region, genu, splenium (edema), thick CC, no spinal cord involvement/lactate peak	c.[5C > A]; [413C > G], p.[(Ala2Asp)]; [(Pro138Arg)]

Bl: blood; CC: corpus callosum; CSF: cerebrospinal fluid; d: day(s); F: families; FD: feeding difficulties; m: month(s); MRI: Magnetic resonance imaging; NA: not available; PMR: psycho-motor regression; PV: periventricular; ur: urinary; WM: white matter; y: year(s).

**Table 5:** Reported patients with MMDS type 5.

Ref.	Nb of Patients (Nb and Origin of Families)	Age at Onset/Age at Death	Metabolic Findings	Impaired Mitochondrial Proteins	Ophthalmic Findings	Clinical Feature	Brain MRI Spectroscopy	Genotype (*ISCA1*:NM_030940.3)
[95]	4 (2F, India)	Neonate (2/4) 2–3 m (2/4)/11 m to 5 y	Elevated CPK (1/2)	NA	Nystagmus (1/4) fundus pigmentation (1/2)	Major PMDD, SP, SE, FD	Pachygyria (2/4), VMG, cerebral and cerebellar WM LD/lactate peak	c.[259G > A]; [259G > A], p.[(Gln87Lys)]; [(Gln87Lys)]
[97]	1 (Italia)	3 m/11 y	High lactate (bl) and glycine (ur) levels	Complexes II, III, and IV, ATP synthesis, lipoylated proteins	*Nystagmus*	Mild PMDD, SP, FD	Vacuolating LD, TCC	c.[29T > G]; [29T > G], p.[(Val10Gly)]; [(Val10Gly)]
[96]	1 (India)	3 m/NA	NA	NA	No	Major PMDD, SP, SE	Pachygyria (*n* = 2/4), VMG, cerebral and cerebellar WM LD/lactate peak	c.[259G > A]; [259G > A], p.[(Gln87Lys)]; [(Gln87Lys)]
[98]	1 (Egypt)	20 m/alive at 7 y	High lactate (bl) level	Complexes I, II and IV, lipoylated proteins	No	Mild PMDD, SP, hemiparesis	Vacuolating LD, TCC, VMG/lactate peak	c.[302A > G]; [302A > G], p.[(Tyr101Cys)]; [(Tyr101Cys)]

Bl: blood; CPK: creatine phospho kinase; F: families; FD: feeding difficulties; LD: leukodystrophy; m: month(s); MRI: Magnetic resonance imaging; NA: not available; PMDD: psycho-motor developmental delay; SE: seizure; SP: spasticity; TCC: thin corpus callosum; ur: urines; VMG: ventriculomegaly; WM: white matter; y: year(s).

## Data Availability

Not applicable.
